# Myelin Formation by Oligodendrocytes Is Enhanced Through Laminin‐411 and Its Derived Peptide

**DOI:** 10.1002/glia.70027

**Published:** 2025-05-08

**Authors:** Binri Sasaki, Momo Oishi, Tomoka Aoki, Mai Hyodo, Chinami Onchi, Nanako Yamada, Hitomi Misawa, Momona Yamada, Chikako Hayashi, Kiyotoshi Sekiguchi, Keisuke Hamada, Yuji Yamada, Yamato Kikkawa, Motoyoshi Nomizu, Nobuharu Suzuki

**Affiliations:** ^1^ Department of Clinical Bioanalysis and Molecular Biology, Graduate School of Medical and Dental Sciences Institute of Science Tokyo/Tokyo Medical and Dental University (TMDU) Tokyo Japan; ^2^ Department of Molecular and Cellular Biology, Graduate School of Medical and Dental Sciences TMDU Tokyo Japan; ^3^ Division for Matrixome Research and Application Institute for Protein Research, Osaka University Osaka Japan; ^4^ Department of Clinical Biochemistry, School of Pharmacy Tokyo University of Pharmacy and Life Science Tokyo Japan

**Keywords:** laminin‐411, myelin, oligodendrocyte

## Abstract

In the central nervous system, oligodendrocytes (OLs) form myelin sheaths that accomplish the efficient transmission of nerve conduction for optimal motor and cognitive functions. OL development and differentiation are regulated by a variety of molecules, including extracellular matrix (ECM) proteins. ECM proteins are also useful as substrates for OL culture. However, the functions of ECM proteins in OL development and myelination remain unclear, and only a limited number of ECM proteins have been characterized and used in in vitro experiments. Here, we investigated the expression and function of laminin (LM) isoforms in OL differentiation and myelination. We found that LM α1, α2, and α4 chains were expressed around blood vessels at the stage of myelination in mice. Functional analyses using recombinant proteins of LM isoforms containing α1, α2, and α4 chains revealed that LM411 and LM411E8, the integrin binding domain of LM411, possessed significant activities in myelin membrane formation of OLs. Furthermore, the peptide A4G47 derived from LM411E8 promoted the activity, which provides evidence of the first peptide in OL myelin formation from ECM proteins. Our findings facilitate a better understanding of ECM functions in OL biology and the development of a new material in OL myelination.

## Main Points

- Laminin (LM) α1, α2, and α4 chains are expressed at the myelination stage of oligodendrocyte (OL).
- LM411 and its E8 fragment enhance OL myelin membrane formation.
- A4G47 from LM411E8 is a novel peptide that promotes OL myelin formation.

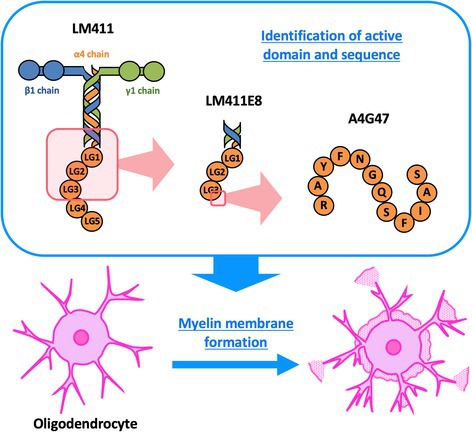

## 1 Introduction

Oligodendrocytes (OLs) are glial cells that form myelin sheaths around neuronal axons in the central nervous system (CNS). Since the main component of the myelin sheath is lipids, such as galactocerebroside (GalC) and cholesterol, myelin functions as an electrical insulator for the rapid conduction of nerve action potentials and maintains optimal motor and cognitive functions (Stadelmann et al. 2019). The development and differentiation of OLs are regulated by various intracellular and extracellular molecules, including extracellular matrix (ECM) proteins (Bauch and Faissner 2022; Yamada et al. 2022; Yellajoshyula et al. 2022; Ghorbani et al. 2024). In addition, OLs and their precursor cells (oligodendrocyte precursor cells: OPCs) are the main producers of ECM proteins in the CNS (Garwood et al. 2004; Song et al. 2018; Yellajoshyula et al. 2021; Elbaz et al. 2024). However, there are still unveiled functions of ECM proteins and their mechanisms during OL development and myelination. Also, ECM proteins are useful as substrates to culture OLs for the evaluation of OL functions and myelination in vitro. But only limited ECM proteins have been characterized in in vitro assays and used for the experiments.

Laminin (LM) is an ECM protein that has cell adhesion activity in basement membranes. LM is a heterotrimeric protein composed of α, β, and γ chains. Five α chains (α1‐5), four β chains (β1‐4), and three γ chains (γ1‐3) have been identified, and various LM isoforms are generated depending on their combinations (Yao 2017). For example, LM111 is composed of α1, β1, and γ1 chains. The five LM G domain‐like (LG) modules (LG1‐5) at the C‐terminus of the α chain have binding activity to cellular receptors. An elastase digestion of LM produces the E8 fragment, containing LG1‐3, which binds to integrins, and the E3 fragment, consisting of LG4‐5 that binds to α‐dystroglycan (α‐DG), sulfatide, and heparin/heparan sulfate proteoglycans (HSPGs) (Suzuki et al. 2005). We have completed the screening of cell binding sequences in 5 of LM α chains using large sets of synthetic peptides (Nomizu et al. 1995; Kato et al. 2002; Makino et al. 2002; Okazaki et al. 2002; Suzuki et al. 2003; Suzuki et al. 2010). The identified active peptides are useful for various applications, such as neurite outgrowth, angiogenesis, wound healing, and drug delivery (Kato et al. 2002; Ikemoto et al. 2006; Mochizuki et al. 2007; Negishi and Nomizu 2019). However, an active peptide that promotes OL differentiation and myelination has not been identified yet.

In the CNS, the gene deletion of LM α2 chain inhibits the survival of OPCs and delays myelination in mice (Relucio et al. 2012). In addition, mutant *dy*/*dy* mice, whose expression of LM α2 chain is deficient, display hypomyelination in the corpus callosum, but not in the spinal cord (Chun et al. 2003). A number of studies used LM211 as a culture substrate for OLs, as well as poly‐D‐lysine (PDL) (Colognato et al. 2004; Eyermann et al. 2012; Ly et al. 2018). Further, Kang and Yao have recently demonstrated that OL‐expressing LM γ1 chain, a component of most LM isoforms, is required for the integrity of the blood–brain barrier (BBB) and myelination by OLs (Kang and Yao 2024). However, characterization of the other LM isoforms in OL development/myelination and in vitro experiments using OLs has not been clarified. We recently reported that LM α1, α2, α4, and α5 chains were expressed in the newborn mouse brain, and E8 fragments of LM211, LM411, and LM511 promoted OPC migration, while those of LM411 and LM511 suppressed apoptosis of OPCs (Suzuki et al. 2019). In this study, we aimed to investigate the expression and function of LM isoforms in OL differentiation and myelination. We found that LM α1, α2, and α4 chains were expressed around blood vessels in the white matter tissue of the murine brain at postnatal day (P)16, when OLs actively myelinate axons. In addition, functional analysis using recombinant proteins of LM isoforms (LM111, LM211, and LM411), containing α1, α2, or α4 chains, revealed that LM411 is remarkably active in OL survival and myelin membrane formation, rather than LM211 and PDL. Furthermore, LM411E8 promoted myelin membrane formation. We finally provide evidence that A4G47, a peptide from LM411E8, promoted OL differentiation and myelin membrane formation. Our findings will contribute to a better understanding of ECM/LM function in OL biology and to the establishment of a new culture method for OLs, on behalf of the currently used culture substrates for OLs, PDL and LM211, or of a new biomaterial for OL myelination.

## 2 Materials and Methods

### 2.1 Animals

P1 and P2 rats and P14 and P16 mice were used for experiments. Mice and rats were anesthetized with isoflurane or an injection of the mixed agent of medetomidine hydrochloride (ZENOAQ) (for mice: 0.75 mg/kg; for rats: 0.375 mg/kg), midazolam (NIG) (for mice: 4 mg/kg; for rats: 2 mg/kg), and butorphanol tartrate (Meiji Seika Pharma) (for mice: 5 mg/kg; for rats: 2.5 mg/kg). To euthanize the animals, decapitation for pups and cervical dislocation for adult rats were performed. All procedures for experimental animals were approved by the Institutional Animal Care and Use Committees of Tokyo Medical and Dental University (Animal experiment approval No: A2023‐122C3). All methods were conducted in strict accordance with the approved guidelines of the institutional animal care committees.

### 2.2 Immunohistochemistry

P14 and P16 mice were anesthetized as described above and were perfused with 4% paraformaldehyde (PFA) (FUJIFILM Wako Pure Chemical). Then, brains or spinal cords that were dissected out were fixed with 4% PFA overnight at 4°C and were replaced into gradient sucrose (15% and 30%) at 4°C. After that, the tissues were embedded with O.C.T. compound (Sakura Finetek) and stored at −80°C. The tissues were thinly sliced into 8 μm sections with a cryostat (Leica Microsystems). The tissue sections were air‐dried and post‐fixed with 4% PFA for 10 min at room temperature or cold acetone for 10 min at −20°C for the CD31 staining. Except for the CD31 staining, an antigen retrieval was then performed by immersing the tissue sections in Target Retrieval Solution (Agilent) and heating them in a microwave. Heating was immediately stopped at the moment that the solution started to boil. The sections were incubated in the solution until they cooled down to room temperature. After blocking with Power Block (BioGenex) for an hour at room temperature, the tissue sections were incubated with primary antibodies at 4°C overnight. After washing with phosphate buffered saline (PBS), fluorescently labeled secondary antibodies were added and incubated for 50 min at room temperature. After washing with PBS, the sections were mounted in ibidi Mounting Medium (ibidi) with DAPI solution (Dojindo) to stain nuclei. The samples were observed with a fluorescence microscope BZ‐X700 (Keyence). Used antibodies are as follows: rabbit anti‐LM (Merck: L‐9393, 1:30 dilution), rat anti‐CD31 (BD Biosciences: 550274, 1:100 dilution), mouse anti‐adenomatous polyposis coli (APC, clone CC‐1; Merck: OP80, 1:100 dilution), mouse anti‐β4 tubulin (Abcam: ab11315, 1:200 dilution), FluoroMyelin Red Fluorescent Myelin Stain (ThermoFisher Scientific: F34652, 1:300 dilution), mouse anti‐Neurofilament (Merck: N5389, 1:200 dilution), rat anti‐LM α chains (Mouse Basement Membrane Bodymap: http://dbarchive.biosciencedbc.jp/archive/matrixome/bm/home.html; α1: 5B7‐H1, 1:200 dilution; α2: 4H8‐2, 1:200 dilution; α3: M3N9‐D10, 1:200 dilution; α4: M49‐N7‐F3, 1:200 dilution; α5: M5N8‐C8, 1:200 dilution), goat anti‐rat IgG‐Alexa Flour 594 (ThermoFisher Scientific: A11007, 1:250 dilution), goat anti‐rabbit IgG‐Alexa Flour 488 (ThermoFisher Scientific: A11034, 1:250 dilution), goat anti‐rabbit IgG‐Alexa Flour 594 (ThermoFisher Scientific: A11037, 1:250 dilution), goat anti‐mouse IgG‐Alexa Flour 488 (ThermoFisher Scientific: A11029, 1:250 dilution), goat anti‐mouse IgG‐Alexa Flour 647 (ThermoFisher Scientific: A21236, 1:250 dilution), and goat anti‐mouse IgG‐Alexa Flour 594 (ThermoFisher Scientific: A11032, 1:250 dilution). In all immunohistochemical analyses, triplicate independent experiments gave similar results. Also, the specificity of antibodies was validated as previously reported (Suzuki et al. 2012; Hayashi et al. 2020b).

### 2.3 Cell Culture Preparation

From P1 or P2 rat pups that were euthanized as described above, cerebral cortexes were dissected out, and then minced with a scalpel and enzymatically digested using papain (Worthington Biochemical) and DNase I (Merck) for 20 min at 37°C. After centrifuging for 5 min at 800 rpm, the supernatant was removed, and a cell pellet was suspended with DMEM20S. The cell suspension was passed through a cell strainer (pore size: 70 μm; SPL life sciences). At last, 1 × 10^7^ cells were seeded per 75 cm^2^ flask (ThermoFisher Scientific). After an incubation for 5 days at 37°C, the culture flasks were shaken at 160 rpm on an orbital shaker for an hour at 37°C. Then, the medium mainly containing microglia was removed and the flasks with fresh DMEM20S were shaken again at 240 rpm for 22 h at 37°C. The medium was collected and let pass through a 70 μm cell strainer. The passed medium was transferred to bacterial‐grade petri dishes (TGK). After an incubation for 15 min at 37°C to let contaminated astrocytes attach to the dish, the medium mainly containing OPCs was collected and centrifuged at 800 rpm for 10 min. The supernatant was carefully removed, and the pellet was suspended with OPC medium. The purity of OL lineage cells in the culture was over 80% in an assessment by immunostaining of their markers.

DMEM20S: 4 mM L‐glutamine (Merck), 1 mM pyruvate (Merck), 20% fetal bovine serum (FBS; MP Biomedicals), and 100 U/mL penicillin and 100 μg/mL streptomycin (ThermoFisher Scientific) in Dulbecco's Modified Eagle Medium (DMEM; high glucose, no glutamine; ThermoFisher Scientific).

Basal chemically defined medium (BDM): 4 mM L‐glutamine, 1 mM pyruvate, 0.1% bovine serum albumin (BSA; Merck), 50 μg/mL apo‐transferrin (Merck), 5 μg/mL insulin (Merck), 30 nM sodium selenite (Merck), 10 nM D‐biotin (Merck), 10 nM hydrocortisone (Merck), and 100 U/mL penicillin, and 100 μg/mL streptomycin in DMEM.

OPC medium: BDM containing 10 ng/mL platelet‐derived growth factor‐AA (ThermoFisher Scientific) and 10 ng/mL basic fibroblast growth factor (ThermoFisher Scientific).

### 2.4 Recombinant Proteins

Recombinant LM111, LM211, and LM411 from VERITAS were used for the experiments. Recombinant LME8 fragments were prepared as described previously (Suzuki et al. 2019). Regarding recombinant LMαE3 fragments (LG4‐5 modules), we used expression plasmids as in the previous report (Hozumi et al. 2009). The LMαE3 proteins with human IgG‐Fc tag added to the C‐terminus were secreted by the LMγ2 signal peptide. After a transfection of the plasmid into 293 free‐style cells using 293fectin Reagent (ThermoFisher Scientific) and culture for 2 days, the conditioned medium was collected, and it was centrifuged for 5 min at 3000 rpm. Complete mini EDTA‐free (Merck) was added to the collected supernatant, and the conditioned medium was passed through a 0.22 μm‐sterilized filter (Merck). Protein G‐Sepharose (GE Healthcare) was washed with PBS and added to the medium. Then, it was mixed using a rotating shaker for 20 h at 4°C. The secreted LMαE3 proteins were captured with Protein G‐Sepharose. Further, the suspension was centrifuged for 5 min at 3000 rpm, and the supernatant was removed. Then, the bound protein to Protein G‐Sepharose was eluted with 0.1 M glycine (pH 2.7) and immediately neutralized with 2 M Tris–Hcl (pH 8.8). We performed Western blotting and Coomassie brilliant blue staining with the samples.

### 2.5 Synthetic Peptides

All peptides were prepared by the 9‐fluorenylmethoxycarbonyl (Fmoc)‐solid phase synthesis with a C‐terminal amide on Rink amide resin (Merck) as described previously (Nomizu et al. 1995). Resulting protected peptide resins were deprotected and cleaved from the resin using trifluoroacetic acid (TFA)–thioanisole–m‐cresol–ethanedithiol–H_2_O (80:5:5:5:5, v/v) at room temperature for 3 h. Crude peptides were precipitated and washed with diethyl ether and then purified by high performance liquid chromatography (HPLC) using a Mightysil RP‐18 column (Kanto Chemical) with a gradient of H_2_O/acetonitrile containing 0.1% TFA. Purity and identity of the peptides were confirmed by HPLC and electrospray ionization mass spectrometry.

### 2.6 Differentiation Assay and Cell Morphology Analysis

The 12‐well slides (MATSUNAMI) were coated with PDL for 30 min at room temperature and were sequentially coated with LM111, LM211, and LM411 (30 nM each) at 4°C overnight. As a control, we prepared only PDL‐coated wells. After washing with PBS, isolated OPCs were plated at 1500 or 3000 cells per well and were cultured with OPC medium for 1 day. Then, the cells were cultured with OL medium for 3 days to induce their differentiation. The cells were fixed with 4% PFA for 10 min at room temperature. The immunostaining method, except only for the step of target retrieval, as described above, was carried out. Used antibodies are as follows: rabbit anti‐NG2 (Merck: AB5320, 1:200 dilution) and mouse anti‐GalC (Merck: MAB342, 1:100 dilution). As shown in Figure 2E, the classification of OL is as follows: Stage 1: less than 6 primary cell processes, Stage 2: more than 5 primary cell processes without myelin‐like membrane sheets, and Stage 3: more than 5 primary cell processes with myelin‐like membrane sheets, which are GalC‐positive areas between multiple cell processes. For cell morphology analysis, LME8 (LM111E8, LM211E8, and LM411E8), LMαE3 (LMα1E3, LMα2E3, and LMα4E3), 10 of cell adhesion active peptides composed of a portion of the amino acid sequence from LMα4LG1–3 modules (shown in Figure 6A and Table 1), and 3 scrambled peptides of A4G47 (shown in Table 2) were similarly analyzed.

**TABLE 1:** Cell attachment active peptides from LM411E8.^a^

Name	Sequence	Region^b^
A4G4	FVLYLGSKNAKK	876–887
A4G6	LAIKNDNLVYVY	892–903
A4G10	AYFSIVKIERVG	924–935
A4G20	DVISLYNFKHIY	1009–1020
A4G24	FFDGSSYAVVRD	1046–1057
A4G25	VVRDITRRGKFG	1054–1065
A4G26	GKFGQVTRFDIE	1062–1073
A4G31	LHVFYDFGFSNG	1102–1113
A4G46	EDSLISRRAYFN	1224–1235
A4G47	RAYFNGQSFIAS	1231–1242

^a^ Ten synthetic peptides, whose amino acid sequences are from LM411E8, were used in this study. These peptides showed cell attachment activity of the human fibrosarcoma cell line HT1080 (Okazaki et al. 2002) and human fibroblasts (Katagiri et al. 2012).

^b^ The regions of the amino acid sequences are indicated by the amino acid numbers in mouse LM α4 chain (Accession number: NP_034811).

**TABLE 2:** Scramble peptides of A4G47.

Name	Sequence^a^
A4G47	RAYFNGQSFIAS
A4G47S1	RAAGSSNFIQFY
A4G47S2	QFSANFARYGIS
A4G47S3	YFSINQASRGFA

^a^ The sequences of the 3 scramble peptides are randomly constituted using the amino acids from A4G47.

OL medium: BDM containing 40 ng/mL triiodothyronine (Merck), 10 ng/mL ciliary neurotrophin factor (ThermoFisher Scientific), and 50 ng/mL N‐acetyl‐L‐cysteine (Merck).

### 2.7 Western Blotting

OLs cultured under the same conditions as the differentiation assay were used as samples. Procedures were performed according to the conditions of the previous study (Hayashi et al. 2020a). The primary antibodies were mouse anti‐CNP (Merck: MAB326, 1:1000 dilution), rabbit anti‐MCT1 (Merck: SAB1305989, 1:400 dilution), and mouse anti‐β‐actin (Fujifilm Wako Pure Chemicals Corporation: 010–27,841, 1:1000 dilution). Secondary antibodies were anti‐mouse IgG antibody‐HRP (Cell Signaling Technology: 7076S, 1:2000 dilution) and anti‐rabbit IgG antibody‐HRP (Cell Signaling Technology: 7074, 1:2000 dilution). As a detection reagent, ImmunoStar LD (Fujifilm Wako Pure Chemical) was used. The intensity of detected protein bands was measured using the Image J software.

### 2.8 Cell Survival Assay

In addition to PDL, recombinant proteins of LM111, LM211, and LM411 (30 nM each) were coated on slide glasses in the same manner as in cell differentiation and morphological analysis. For LM, a well with only PDL coat was prepared as a control. After washing with PBS, OPCs were seeded, and after differentiation induction, they were cultured at 37°C for 24 h. The subsequent detection of apoptosis by the TUNEL method was performed according to the instructions for the In Situ Apoptosis Detection Kit (Takara Bio) used. Subsequent immunostaining was performed in the same manner as the cell differentiation and morphological analysis, and the TUNEL‐positive rate in PLP‐positive cells was measured. The same analysis was performed with LME8 (LM111E8, LM211E8, and LM411E8) and LMαE3 (LMα1E3, LMα2E3, and LMα4E3). Used antibodies are as follows: rabbit anti‐PLP (Abcam: ab28486, 1:100 dilution), secondary antibody: goat anti‐rabbit IgG‐Alexa Fluor 594.

### 2.9 Statistical Analyses

Data are presented as the mean ± SD of three to five independent experiments performed on separate cell preparations. It was difficult to prepare the same differentiation condition of the primary cells between independent culture experiments. Therefore, we set an average of the control as 1.0, and then used relative values of samples to the control for representing the data in each experiment. For the statistics, we analyzed dispersion of the relative values of samples to 1.0 as previously reported (Nogami et al. 2020). All statistical analyses were performed using the R programming software. Comparisons between experimental groups were made by ANOVA followed by a post hoc Tukey's multiple comparison test. The statistical significance was defined as *: *p* < 0.05; **: *p* < 0.01.

## 3 Results

### 3.1 LM in the Perivascular Basement Membrane Interacts With OLs

**FIGURE 1:**
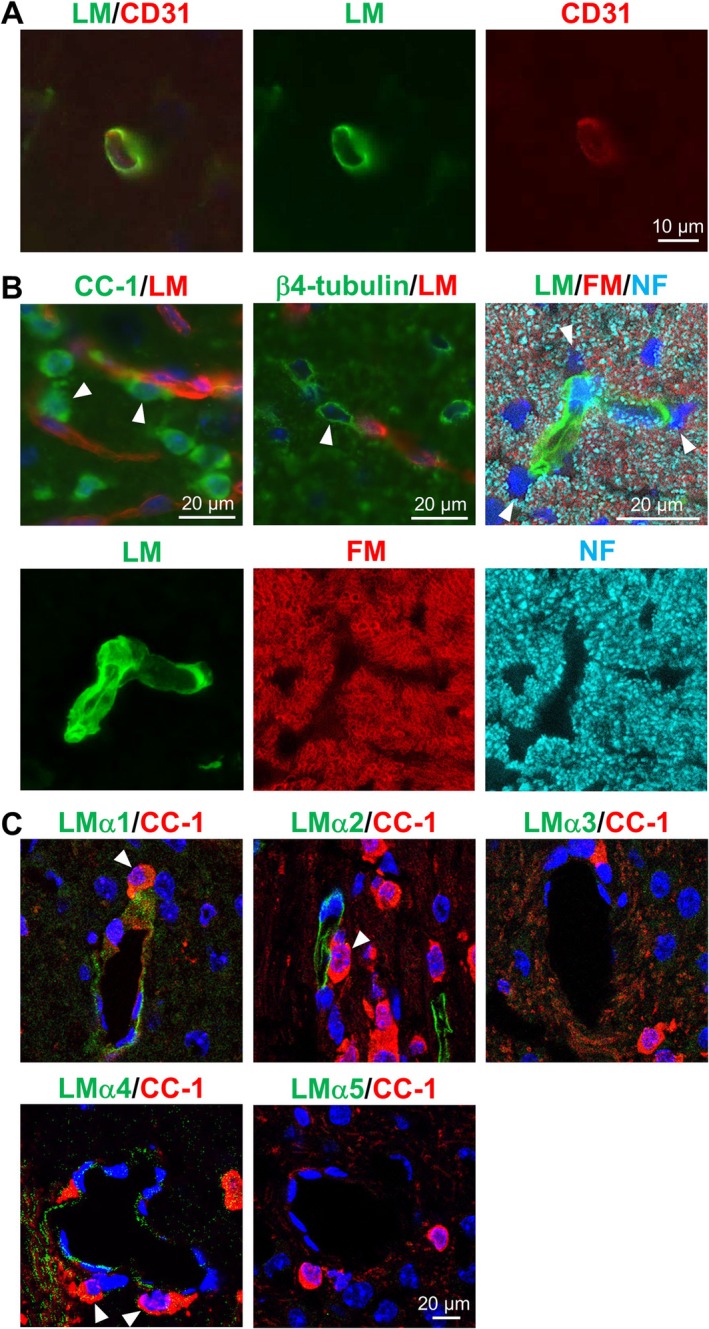
LM expression and perivascular OLs in the CNS tissues. (A) Immunohistochemistry of LM (green) and CD31 (red) in the P14 mouse spinal cord. Scale bars: 10 μm. (B) Left and center upper panels show immunohistochemistry using LM (red) and OL markers (green), CC‐1 and β4‐tubulin. The right upper panel shows triple staining using LM (green), FluoroMyelin (FM; red), and Neurofilament (NF, cyan). Lower panels are single staining images of LM, FM, and NF. DAPI staining (blue) was used to visualize nuclei. Arrowhead: Perivascular OL. Scale bars: 20 μm. (C) Immunohistochemistry of LM α chains (green) and CC‐1 (red) in the P16 WT mouse brain. DAPI staining (blue) was used to visualize nuclei. Arrowhead: OL attached LM. Scale bars: 20 μm.

We first examined the expression of LM and its interaction with OLs in the white matter of P14 mouse spinal cord. In the white matter tissue, LM was expressed in the perivascular basement membrane that surrounded CD31‐positive endothelial cells (Figure 1A). The result of immunohistochemistry with antibodies of APC (clone: CC‐1) and β4 tubulin, markers for OL cell body and OL cell body/process, respectively, showed that there were some mature OLs in contact with the perivascular LM and they spirally extended their processes along the vessels (Figure 1B: left and center upper panels). In addition, a number of myelin rings around these OLs that adhered to the perivascular LM were observed (Figure 1B: right upper panel and lower panels). It is possible that these OLs form myelin adjacent to blood vessels. We then examined the expression of 5 LM α chains. Semi‐quantitative reverse transcription‐PCR detected the mRNA expression of all the LM α chains, particularly α4 at a high level, in the tissue during 1–3 postnatal weeks (Figure S1). However, in immunostaining using their specific antibodies, the protein expression of LM α1, α2, and α4 chains, but not α3 and α5 chains, was found in the perivascular basement membrane of the white matter tissue when OLs actively myelinated axons (Figure 1C). These results, in particular, the immunostaining analyses, suggest that perivascular LM, which is composed of α1, α2, or α4 chains, interacts with OLs that are possibly myelinating axons.

### 3.2 LM411 Promotes Myelin Membrane Formation of OLs

**FIGURE 2:**
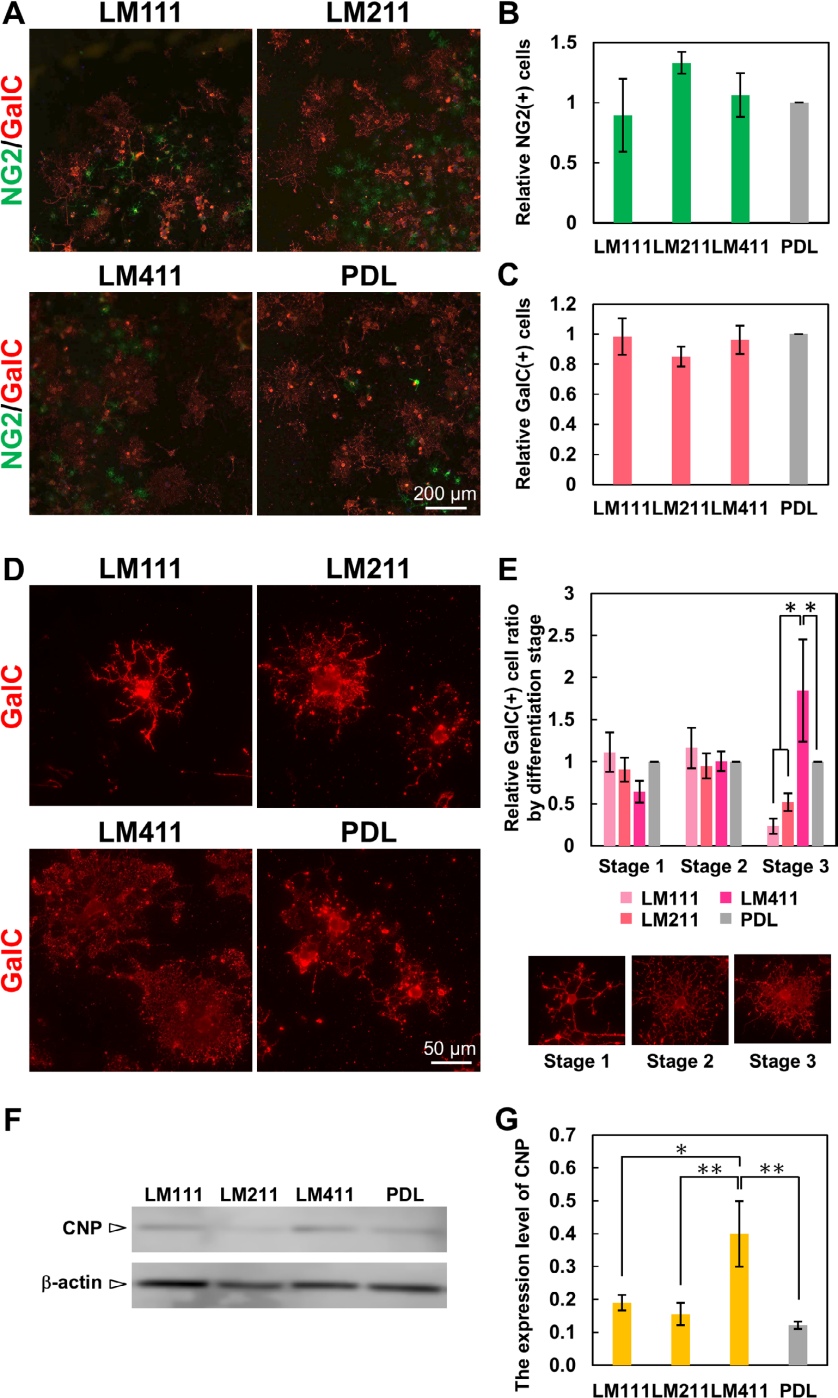
The effect of LM isoforms on morphological differentiation of OLs. (A) Immunocytochemistry of primary OL lineage cells using antibodies against NG2 (green) and GalC (red). Scale bars: 200 μm. (B) Quantification of the percentages of NG2 positive cells. There are no significant differences between LMs and PDL (number of counted cells: 50–200 per condition, *n* = 3). Error bars represent mean ± s.d. (C) Quantification of the percentages of GalC positive cells. There are no significant differences between LMs and PDL (number of counted cells: 50–200 per condition, *n* = 3). Error bars represent mean ± s.d. (D) Morphology of GalC‐positive OLs on LM isoforms. OLs formed sheet‐like myelin membranes on LM411. Scale bars: 50 μm. (E) Samples for morphological classification and quantification of the morphological differentiation classes of GalC positive cells. Error bars represent mean ± s.d. (*: *P* < 0.05, number of counted cells: 100–200 per condition, *n* = 3; Tukey test). (F) Representative images of Western blotting of CNP and β‐actin in OLs on LM isoforms and (G) quantification of the Western blotting analysis are shown. Error bars represent mean ± s.d. (*: *p* < 0.05, **: *p* < 0.01, *n* = 3; Tukey test).

Due to the protein expression pattern of LM α chains in the tissue, we next asked which type of biological activities in OLs were induced by the LMs. Therefore, we analyzed the effect of the LM isoforms that comprise these α chains (LM111, LM211, and LM411) in OL differentiation using primary rat OPCs. We plated OPCs on the LM‐coated wells and induced their differentiation into OLs. PDL‐coated wells were used as a control. When the percentages of NG2‐positive OPCs and GalC‐positive OLs were assessed, there were no significant differences between the 3 LM isoforms and PDL (Figure 2A–C). However, the LM isoforms displayed morphological differences among them. Particularly, LM411 promoted the morphological maturation of OLs, compared with LM111, LM211, and PDL (Figure 2D,E). Some of the OLs on LM411 strikingly increased branched processes (Figure S2). As shown in Figure 2E, OLs increase the number of cell processes as they differentiate and then form sheet‐like myelin membranes (Figure 2E: from Stage 1 to 3). The proportion of stage 3 cells, which were defined by the formation of sheet‐like myelin membranes, was higher in OLs on LM411 compared with LM111, LM211, and PDL (Figure 2D,E). In addition, we analyzed the protein expression level of CNP, a marker for OLs differentiated from OPCs, by Western blotting. As a result, the expression level of CNP protein was significantly increased on LM411 compared to the other LMs and PDL (Figure 2F,G). These results suggest that LM411 promotes the differentiation of OLs and the formation of the myelin membrane.

In vivo, OLs obtain glucose provided from blood vessels through glucose transporter 1 (GLUT1) and supply monocarboxylates, such as pyruvate and lactate, to axons for their homeostasis via monocarboxylate transporter 1 (MCT1) on myelin (Fünfschilling et al. 2012; Lee et al. 2012). We thus examined the expression of MCT1 and GLUT1 in OLs cultured on the LM isoforms. As a result, LM411 increased the MCT1 expression at the tips of OL processes, which is localized in the internode surface of myelin, whereas MCT1 expression on the other LM isoforms was similar to that on PDL (Figure S3A). No obvious expression of GLUT1 was observed on all the LM isoforms, compared to PDL (Figure S3B). The expression level of MCT1 was also analyzed by Western blotting, which showed that the MCT1 expression was significantly increased in OLs cultured on LM411 (Figure S4A,B). From these results, LM411 may play a role in the supply of monocarboxylates from OLs toward axons surrounding blood vessels.

### 3.3 LM411 Suppresses Apoptosis of OLs

**FIGURE 3:**
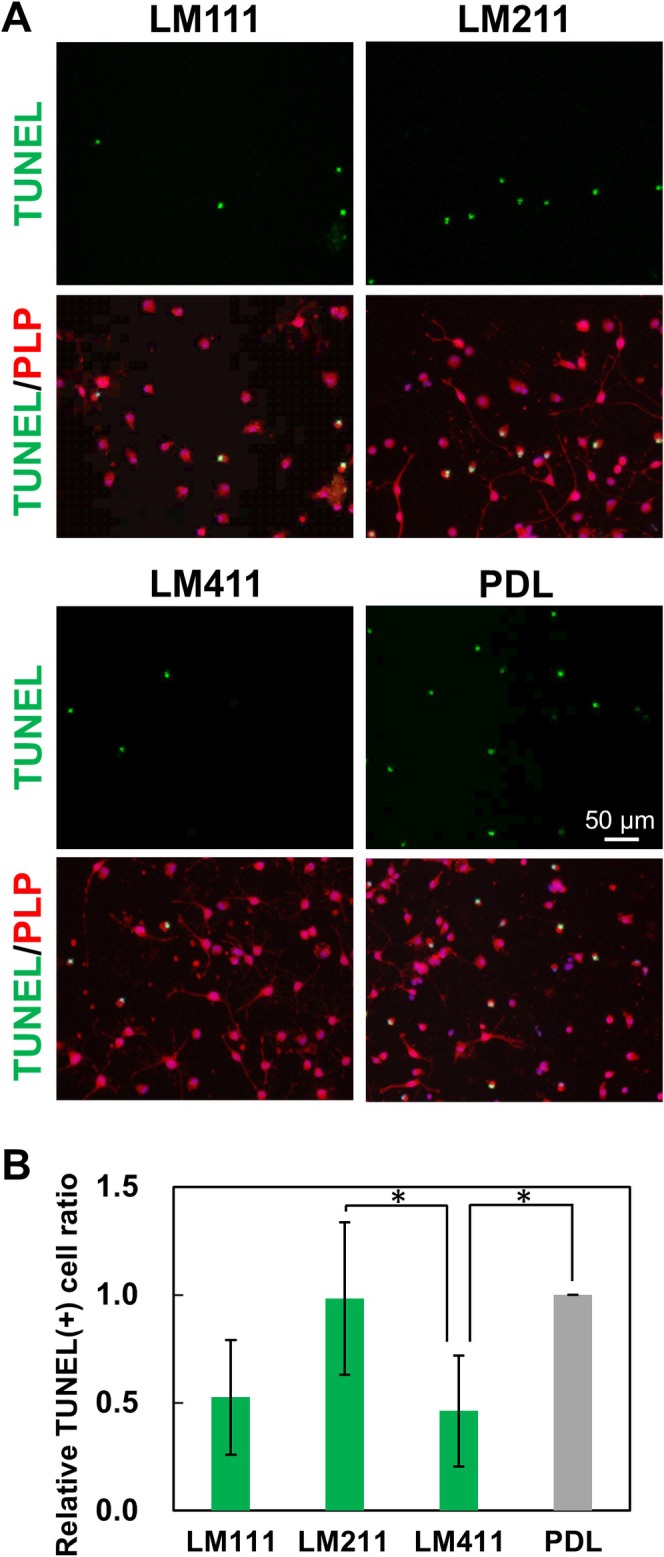
The effect of LM isoforms on OL survival. (A) TUNEL (green) assay with immunocytochemistry of PLP (red) in OL on LM isoforms. OL apoptosis was reduced on LM411. Scale bars: 50 μm. (B) The relative ratio of the number of TUNEL‐positive OLs on the LM isoforms to PDL. Error bars represent mean ± s.d. (*: *p* < 0.05, number of counted cells: 1000–2000 per condition, *n* = 5; Tukey test).

Since LM positively regulates OL survival, we performed the detection of apoptosis on the LM isoforms. We prepared OLs cultured on the LM isoforms; afterwards, detection of TUNEL and PLP, apoptosis and OL markers, respectively, was carried out. As a result of measuring the TUNEL positive rate in PLP‐positive cells, it was significantly lower in LM411 compared to LM211 and PDL (Figure 3A,B). This indicates that LM411 reduces apoptosis and promotes the survival of OLs.

### 3.4 LM411E8 Promotes Myelin Membrane Formation of OLs

**FIGURE 4:**
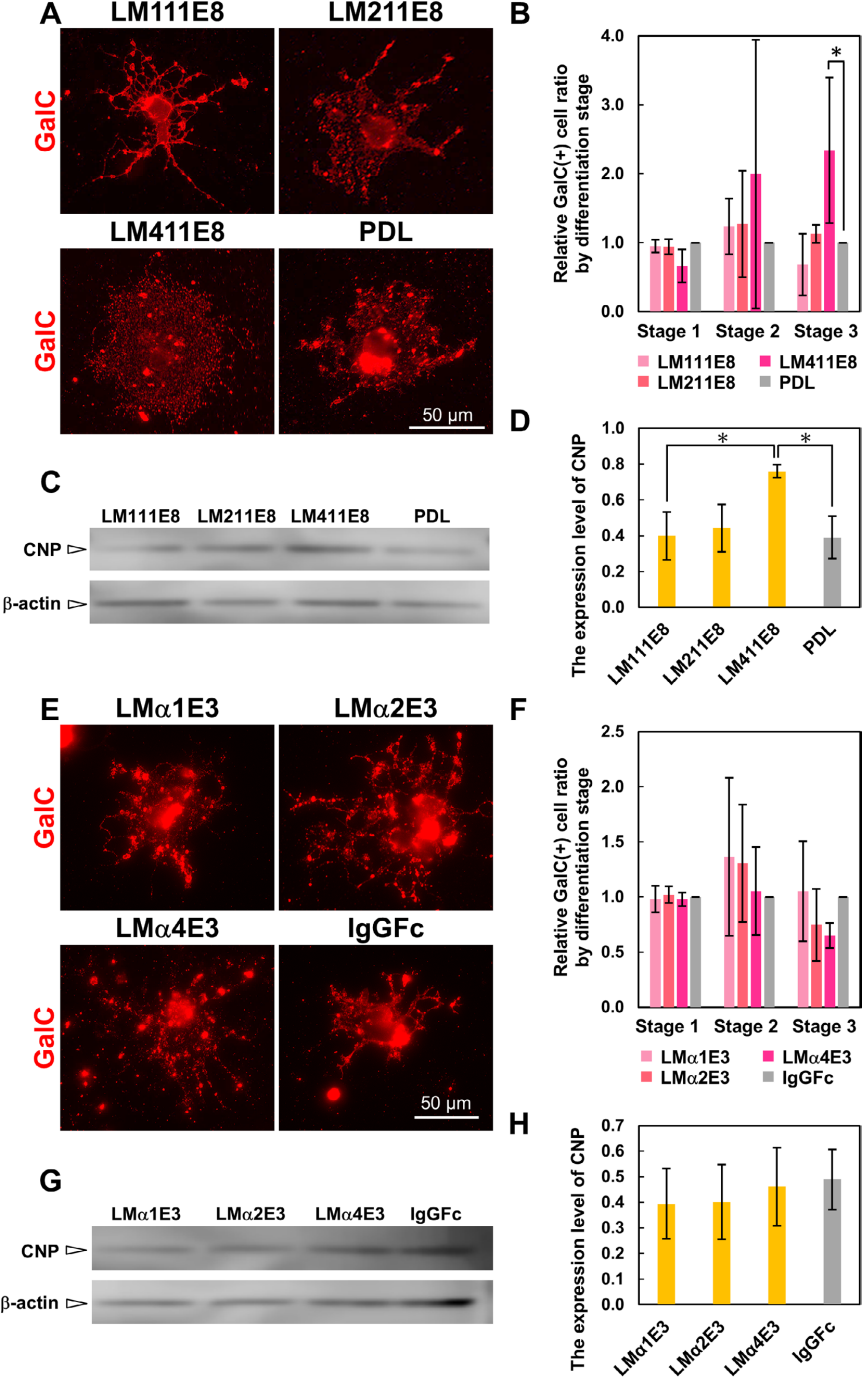
The effect of LME8 and LMαE3 fragments on morphological differentiation of OLs. (A) Immunocytochemistry of GalC (red) in OLs on the LME8 fragments. OLs formed sheet‐like myelin membranes on LM411E8. Scale bars: 50 μm. (B) Quantification of the morphological differentiation classes of GalC‐positive cells on LME8s. Error bars represent mean ± s.d. (*: *p* < 0.05, number of counted cells: 50–100 per condition, *n* = 4; Tukey test). (C) Representative images of Western blotting of CNP and β‐actin in OLs on LME8 fragments and (D) quantification of the Western blotting analysis are shown. Error bars represent mean ± s.d. (*: *p* < 0.05, *n* = 3; Tukey test). (E) Immunocytochemistry of GalC (red) in OL on LMαE3 fragments. No difference was observed between LMαE3s. Scale bars: 50 μm. (F) Quantification of the morphological differentiation classes of GalC‐positive cells on LMαE3s. Error bars represent mean ± s.d. (number of counted cells: 50–100 per condition, *n* = 3). (G) Representative images of Western blotting of CNP and β‐actin in OLs on LMαE3 fragments and (H) quantification of the Western blotting analysis are shown. Error bars represent mean ± s.d. (*n* = 3).

Further, we attempted to identify the active domain of the LM isoforms for the morphological differentiation of OLs. Recombinant proteins of E8 (LM111E8, LM211E8, and LM411E8) and E3 (LMα1E3, LMα2E3, and LMα4E3) were used for the analyses in the same assays as LMs. IgGFc was used as a control for LMαE3 since recombinant LMαE3s were fusion proteins with IgGFc. As a result of evaluating the morphological maturation of OLs, we found that for E8, there was a significantly higher number of OLs with sheet‐like myelin membrane formation on LM411E8 (Figure 4A,B). On the other hand, no significant difference in morphological differentiation was observed between LMαE3s (Figure 4E,F). Also, a Western blotting analysis revealed that the expression of CNP was increased in OLs cultured on LM411E8, compared with LM111E8 and PDL (Figure 4C,D). There were no significant differences between LMαE3s and the control (Figure 4G,H). These results indicate that LM411E8 is the active domain of LM411 in promoting the differentiation of OLs.

### 3.5 LM411E8 and LMα4E3 Do Not Suppress Apoptosis of OLs

**FIGURE 5:**
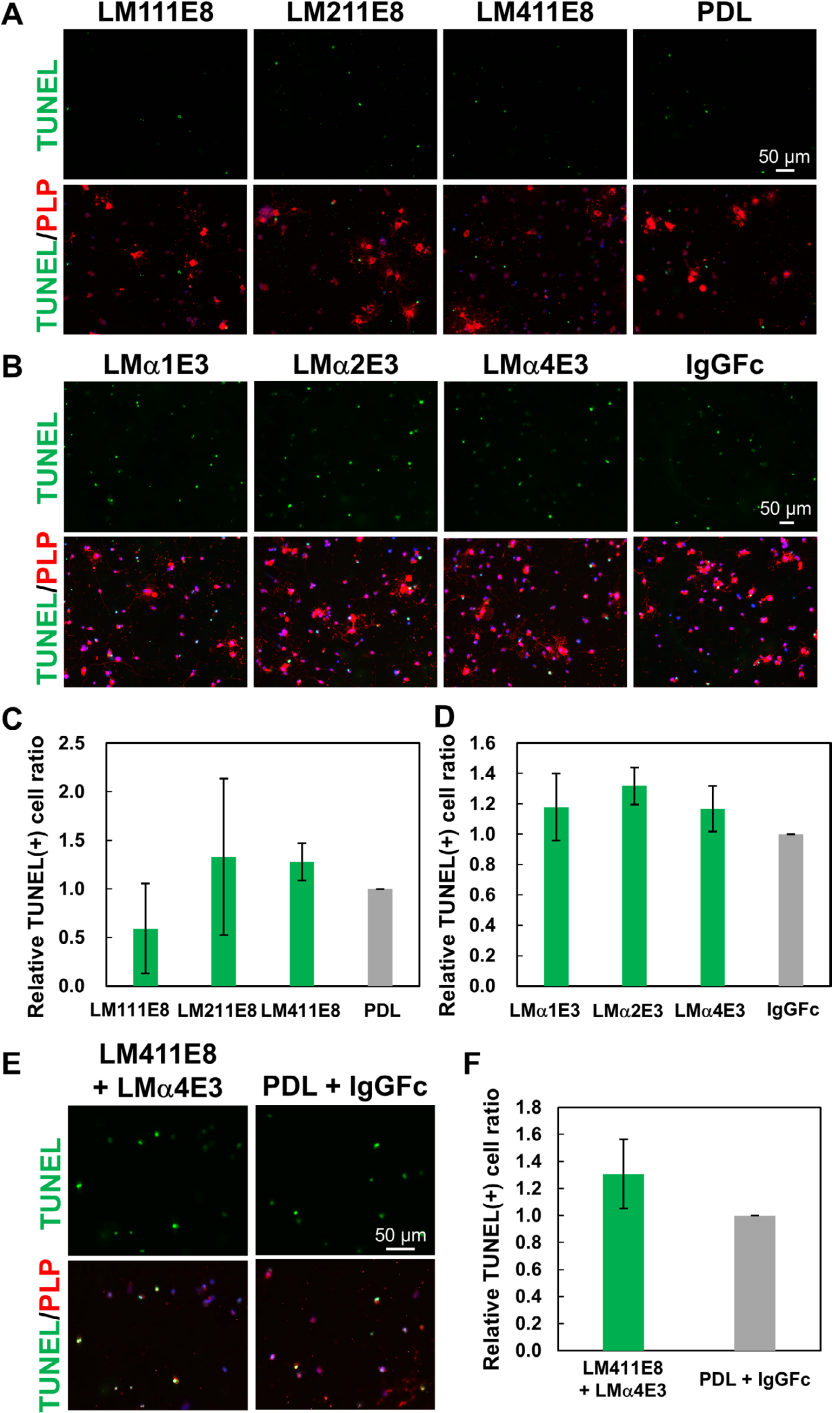
The effect of LME8 and LMαE3 fragments on apoptosis of OLs. TUNEL assay (green) with immunocytochemistry of PLP (red) in OL on (A) LME8s and (B) LMαE3s. There were no obvious differences between the E8 and E3 fragments of LMs. Scale bars: 50 μm. The relative ratio of the number of TUNEL‐positive OLs on (C) LME8s to PDL and (D) LMαE3 to IgGFc. (E) TUNEL assay (green) with immunocytochemistry of PLP (red) in OL on the combination of LM411E8 with LMα4E3. (F) The relative ratio of the number of TUNEL‐positive OLs on LM411E8/LMα4E3 to the control PDL/IgGFc. Error bars represent mean ± s.d. (number of counted cells: 1000–2000 per condition, *n* = 3).

Since the active domain of LM in OL morphological differentiation was determined, we similarly evaluated each LM E8 and E3 fragment for OL survival. Unlike morphological differentiation, there was no difference in the TUNEL positive rate between the LME8s (Figure 5A,C) and LMαE3s (Figure 5B,D), and no significant cell survival activity was observed. Furthermore, the investigation into whether the survival of OLs is promoted under the condition in which both LM411E8 and LMα4E3 are present revealed that the combination of LM411E8 with LMα4E3 did not reduce the number of apoptotic cells (Figure 5E,F). From these observations, only LM411E8, LMα4E3, or the combination is not sufficient to induce the survival signaling of LM411 in OLs.

### 3.6 The A4G47 Peptide Promotes Myelin‐Like Membrane Formation of OLs

**FIGURE 6:**
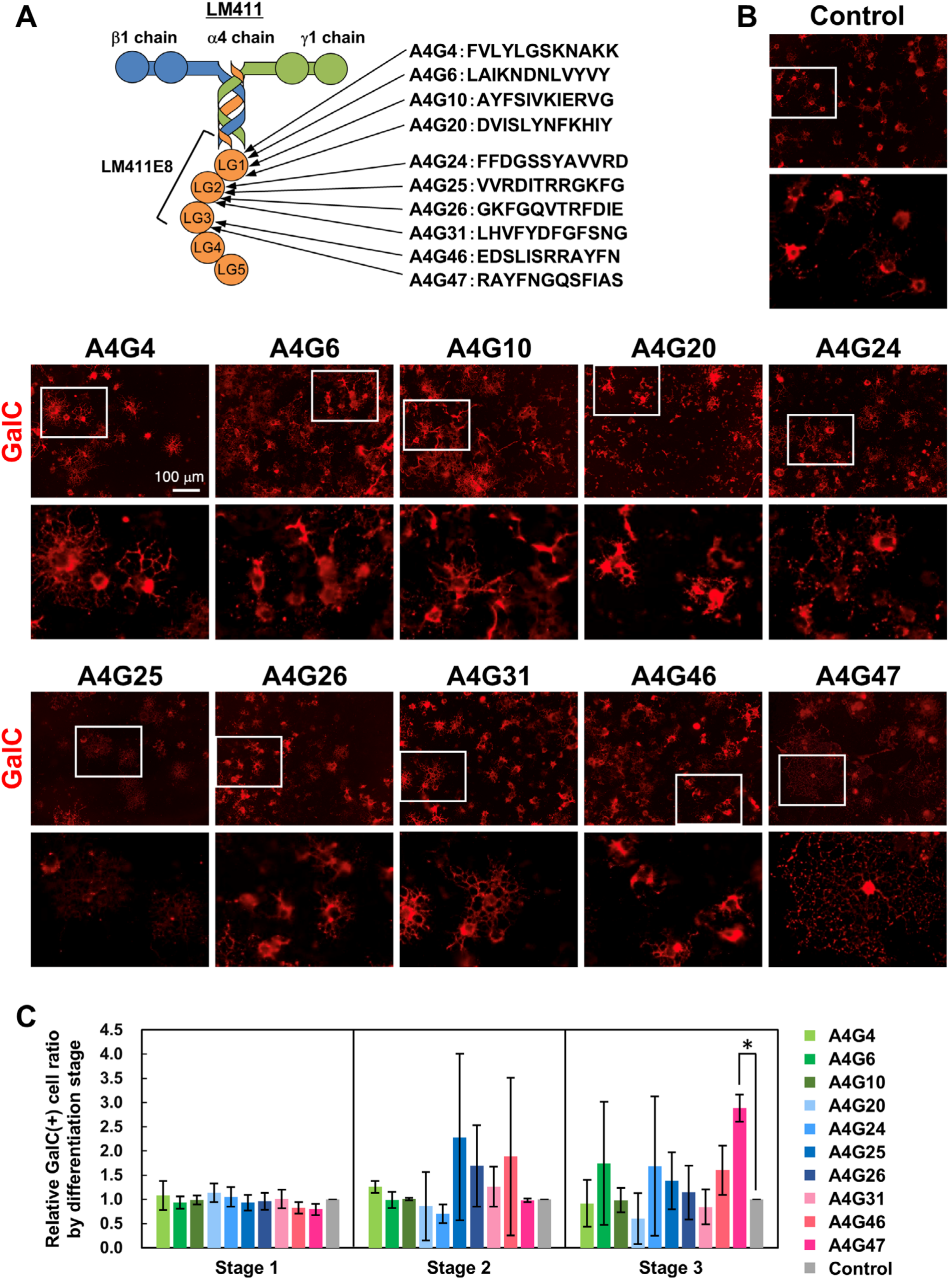
The effect of peptides composed of a portion of the amino acid sequence of LM411E8 on morphological differentiation of OLs. (A) Ten cell adhesive peptides derived from the amino acid sequence of the LM α4 LG1‐3 modules. (B) Immunocytochemistry of GalC (red) in OLs on each peptide. OLs formed sheet‐like myelin membranes on A4G47. Scale bars: 100 μm. The lower panels are high magnification images of the white boxes of the upper panels. (C) Quantification of the morphological differentiation classes of GalC‐positive cells on each peptide. Error bars represent mean ± s.d. (*: *p* < 0.05, number of counted cells: 100–200 per condition, *n* = 3; Tukey test).

From the result that LM411E8 had the activity of promoting myelin membrane formation of OLs, we investigated the activity of 10 types of cell adhesive peptides derived from the LMα4LG1–3, which is the main region of binding to cellular receptors in LM411E8 (Figure 6A and Table 1), in OL differentiation. As a result of culturing OLs on wells coated with the peptides, 9 out of the 10 peptides showed a similar level of morphological differentiation as the control PDL. However, A4G47 exhibited a higher number of sheet‐like myelin membrane‐forming OLs (Figure 6B). By measuring the number of cells in each Stage 1 to Stage 3 with GalC‐positive OLs, there was a significantly higher number of Stage 3 OLs on A4G47 (Figure 6C). These results suggest that the morphological differentiation of OLs was prominently promoted by A4G47.

### 3.7 The Amino Acid Sequence of A4G47 Is Specifically Required for Its Activity

**FIGURE 7:**
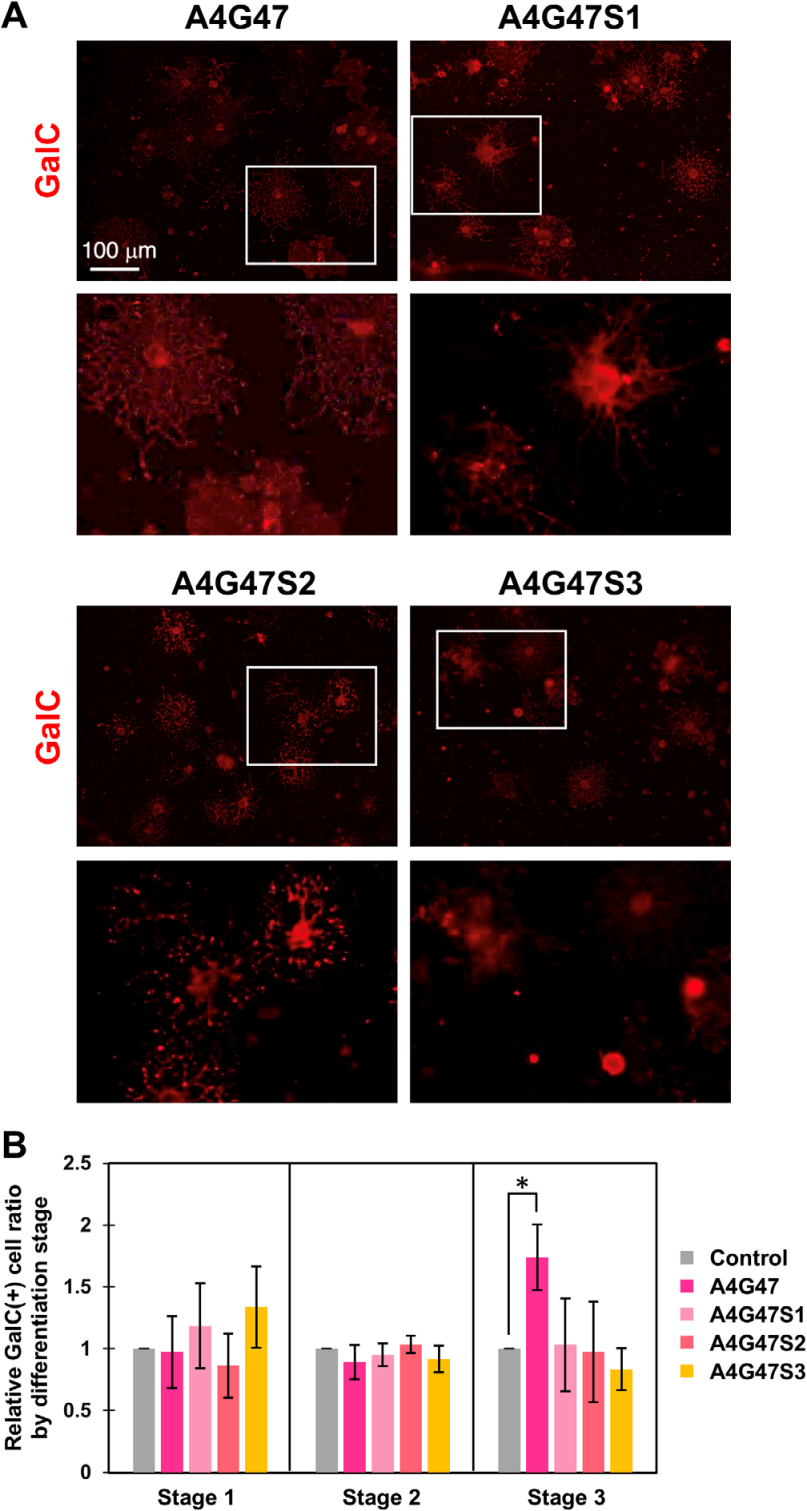
The effect of the change of amino acid sequence of A4G47 on morphological differentiation of OLs. (A) Immunocytochemistry of GalC (red) in the OL on the A4G47 and three synthetic peptides in which the amino acid sequences of A4G47 were randomly rearranged. The scramble peptides did not show the activity of myelin formation. The lower panels are high magnification images of the white boxes of the upper panels. Scale bars: 100 μm. (B) Quantification of the morphological differentiation classes of GalC‐positive OLs on each peptide. Error bars represent mean ± s.d. (*: *p* < 0.05, number of counted cells: 50–100 per condition, *n* = 5; Tukey test).

To examine whether the activity of A4G47 in promoting myelin membrane formation is dependent on its amino acid sequence, we prepared 3 types of synthetic peptides, A4G47S1, A4G47S2, and A4G47S3, in which amino acids contained in A4G47 are randomly reconstituted (Table 2). Using the random sequence scramble peptides, the activity in the morphological maturation of OLs was tested, and the activity of promoting myelin membrane formation of OLs, as seen in A4G47, was not observed in the scramble peptides (Figure 7A,B). These results indicate that the specific amino acid sequence of A4G47 is required for its activity to promote myelin membrane formation of OLs.

## 4 Discussion

In this study, we found that LM α1, α2, and α4 chains were expressed in the perivascular basement membrane at the age of 2 weeks, when myelination by OLs is peaked in the murine CNS tissue. Among these 3 LM isoforms, the differentiation and survival of OLs were significantly promoted on LM411, compared with LM111, LM211, and PDL. The promoting activity of LM411 in myelin membrane formation was exerted in the E8 domain of LM411. In addition, the peptide A4G47 from LM411E8 showed the similar activity as LM411E8. To our knowledge, this is the first report that demonstrated the marked function of LM411 in OL biology and that identified a peptide to promote myelin membrane formation from ECM proteins.

Among LM isoforms, LM211 has been well‐characterized in OL development and myelination. The deficiency of the gene function of the LM α2 chain in mice displays defects in OPC survival and myelination (Chun et al. 2003; Relucio et al. 2012). In in vitro studies, LM211 promotes OL survival and process formation through the interaction with the cell surface receptors, including integrin α6β1, sulfatide, and α‐DG (Colognato et al. 2002; Eyermann et al. 2012; Baron et al. 2014; Ly et al. 2018). Further, the signaling downstream of LM211 binding to the receptors regulates the development of OL lineage cells, in cooperation with the signaling of growth factors, such as platelet‐derived growth factor, insulin‐like growth factor‐1, and neuregulin‐1 (Colognato et al. 2004; Galvin et al. 2010). Regarding the analysis of the other LM isoforms, we previously reported that LM α1, α2, α4, and α5 chains were expressed in the perivascular basement membrane in the brain tissue of newborn mice, when OPCs are not yet differentiated into OLs (Suzuki et al. 2019). Also, OPCs attach to LM211E8, LM411E8, and LM511E8, and the apoptosis of OPCs is suppressed by LM411E8 and LM511E8, though their migration was elevated on LM211E8, LM411E8, and LM511E8 (Suzuki et al. 2019). In this study, we found that LM411 and LM411E8 exerted more significant activities in the differentiation and survival of OLs, compared with the other LMs and their E8s. The difference between the LM isoforms in the activities is presumably due to interactions with different sets of cellular receptors. The binding affinity to integrin α6β1 of LM411 is higher than those of LM111 and LM211, while integrin α7β1, sulfatide, and α‐DG more selectively interact with LM111 and LM211 than LM411 (Talts et al. 1999, 2000; Kortesmaa et al. 2000; Nishiuchi et al. 2006; Yamada and Sekiguchi 2015). The differences in the combination and binding affinities of these receptors may result in the distinct activities between the LM isoforms. OLs probably express the receptors with the combination, which preferentially interact with LM411. In particular, a coordination of the integrin signaling should be more important in myelin membrane formation because the receptors for LM411E8 are integrins, rather than the other receptors. In terms of OL survival on LM411, neither LM411E8, LMα4E3, nor the combination of both of them showed the activity, indicating that the other region(s) of LM411 or the protein conformation of LM411E8‐E3 as a single polypeptide is(are) necessary for interaction with the receptors on OLs.

In the present study, we identified the peptide A4G47, whose sequence is derived from the LG3 module of the LM α4 chain. To our knowledge, A4G47 is the first peptide to promote myelin membrane formation from ECM proteins. We previously prepared LM‐derived peptide‐conjugated chitosan membranes, which are a biodegradable polymer, and found their positive effectiveness in neurite outgrowth, angiogenesis, and wound healing (Kato et al. 2002; Ikemoto et al. 2006; Mochizuki et al. 2007). A polymer or a carrier protein conjugated with A4G47 may become a useful biomaterial for the promotion of OL myelination in demyelination diseases, as typified by multiple sclerosis, and in injuries accompanied by glial scar, which blocks OL survival and myelination, such as spinal cord injury. Also, it is possible to prepare a better quality of OL culture, which might be useful for a cell transplantation for the diseases and/or the injuries. Our previous study showed that the attachment of human fibroblasts to A4G47 was inhibited by EDTA and heparin, suggesting that both molecules, which require divalent cation for their activation, like integrins, and HSPGs are predicted as receptors for A4G47 (Katagiri et al. 2012). However, LM411E8 does bind to integrins, but does not bind to heparin/HSPGs. In addition, integrins recognize the conformation in 3‐dimensional (D) protein structure of LMs (Arimori et al. 2021), although 10–20 mer peptides, including A4G47, are usually unable to form the type of defined 3D structures. Thus, the A4G47 sequence seems not to fully cover the activity of LM411 and LM411E8. Other receptor binding sites and/or domains that function as a base or an axis to expose those binding sites to integrins on LM411 are probably required for exerting the activity of intact LM411.

Another important question is the in vivo relevance of these activities of LM411. Kang and Yao have recently demonstrated that LM γ1 chain, the most common LM γ subunit, is required for OPC proliferation and OL differentiation, including myelination, as well as the integrity/maintenance of BBB (Kang and Yao 2024). This indicates that LMs in the perivascular basement membrane play an essential role in myelination. In the present study, our result indicated the possibility that OLs attached to vascular LMs formed myelin surrounding axons. In addition, OLs cultured on LM411 increased the expression of MCT1, but not GLUT1. OLs support axonal homeostasis due to supplying monocarboxylates, such as pyruvate and lactate, via MCT1 on the internodal surface of oligodendroglial myelin (Fünfschilling et al. 2012; Lee et al. 2012), whereas they take glucose, which is abundant around blood vessels, as a source of the monocarboxylates through GLUT1 (Saab et al. 2016). It is possible that the interaction between OLs and LM411 is a key event in the supply bridge of glucose/monocarboxylates from blood vessels to axons. However, more active myelination was not observed around blood vessels. There are reports that indicate the distributions of LM expression not only in vascular basement membrane but also in parenchyma and on axonal surface in the CNS tissues (Colognato et al. 2002; Lau et al. 2013). In the distal regions from blood vessels, the non‐basement membrane types of LM411 may exist and promote OL myelination, or other molecule(s) may regulate it on behalf of LM411. Furthermore, the expression of matrix metalloproteinase (MMP)‐9, which cleaves various ECM proteins including LMs, is increased in both the corpus callosum and optic nerve during developmental myelination (Uhm et al. 1998; Oh et al. 1999; Beliveau et al. 2010). Also, cellular process formation is reduced in OLs from MMP‐9‐deficient mice (Oh et al. 1999). Digestion of ECM proteins by the proteinases, including MMP‐9, during OL differentiation may change the conformation and distribution of ECM proteins in the perivascular basement membrane or directly cleave LM411, which results in the exposure of the A4G47 sequence to OL receptors.

We here discovered LM411, LM411E8, and A4G47 as novel OL‐myelin formation proteins or peptides. PDL and LM211 are currently used as common culture substrates for OLs. Our findings showed that LM411, LM411E8, and A4G47 are better tools/materials for OL culture to observe myelin formation. Usage of them in OL culture experiments will give a higher efficiency in myelin membrane formation. Together, our discovery will be useful for a better understanding of OL biology and for the development of materials applicable in OL experiments and studies regarding related diseases/injuries.

## Author Contributions

Y.K., M.N., and N.S. designed the experiments. B.S., M.O., T.A., M.H., C.O., N.Y., H.M., M.Y., C.H., and N.S. performed experiments. B.S., M.O., T.A., N.Y., and H.M. analyzed data. K.S., K.H., and Y.Y. prepared resources. B.S. and N.S. wrote the manuscript. N.S. supervised this work. All the authors read and approved the final manuscript.

## Conflicts of Interest

The authors declare no conflicts of interest.

## Supporting information


**Supplementary Figure S1:** The mRNA expression levels of LM α chains in wild‐type mouse brain.
**Supplementary Figure S2:** The effect of LM411 on morphological differentiation of OLs.
**Supplementary Figure S3:** The effect of LM isoforms on the expression of MCT1 and GLUT1 in OLs.
**Supplementary Figure S4:** The increased expression of MCT1 in OLs on LM411.

## Data Availability

All the data are presented in the manuscript as figures.
