# Supplementary material for: Myelin Formation by Oligodendrocytes Is Enhanced Through Laminin‐411 and Its Derived Peptide
Source: Glia. 2025 May 8;73(8):1692–706. doi: 10.1002/glia.70027 (PMC12185975; doi:10.1002/glia.70027)
Supplement: Supplementary file 1 — Supplementary Figure S1: The mRNA expression levels of LM α chains in wild‐type mouse brain. Supplementary Figure S2: The effect of LM411 on morphological differentiation of OLs. Supplementary Figure S3: The effect of LM isoforms on the expression of MCT1 and GLUT1 in OLs. Supplementary Figure S4: The increased expression of MCT1 in OLs on LM411. [file GLIA-73-1692-s001.pdf]

Supplementary Information

Myelin Formation by Oligodendrocytes Is Enhanced through Laminin-411 and Its Derived Peptide

Binri Sasaki[1], Momo Oishi[2], Tomoka Aoki[1], Mai Hyodo[2], Chinami Onchi[2], Nanako Yamada[1], Hitomi Misawa[1], Momona Yamada[2], Chikako Hayashi[2], Kiyotoshi Sekiguchi[3], Keisuke Hamada[4], Yuji Yamada[4], Yamato Kikkawa[4], Motoyoshi Nomizu[4], and Nobuharu Suzuki[1,2*]

[1]Department of Clinical Bioanalysis and Molecular Biology, Graduate School of Medical and Dental Sciences, Institute of Science Tokyo/Tokyo Medical and Dental University (TMDU), Tokyo, Japan
[2]Department of Molecular and Cellular Biology, Graduate School of Medical and Dental Sciences, TMDU, Tokyo, Japan
[3]Division for Matrixome Research and Application, Institute for Protein Research, Osaka University.
[4]Department of Clinical Biochemistry, School of Pharmacy, Tokyo University of Pharmacy and Life Science, Hachioji, Tokyo, Japan

*To whom correspondence should be addressed: Nobuharu Suzuki, Ph.D., Department of Clinical Bioanalysis and Molecular Biology, Graduate School of Medical and Dental Sciences, Institute of Science Tokyo/TMDU, 1-5-45 Yushima, Bldg 3, Bunkyo-ku, Tokyo, Japan, 113-8510, E-mail: nsuzbb@tmd.ac.jp, Tel: +81-3-5803-5364; Fax: +81-3-5803-5364.

Supplementary Fig. S1: The mRNA expression levels of LM α chains in wild-type mouse brain

Supplementary Fig. S2: The effect of LM411 on morphological differentiation of OLs

Supplementary Fig. S3: The effect of LM isoforms on the expression of MCT1 and GLUT1 in OLs

Supplementary Fig. S4: The increased expression of MCT1 in OLs on LM411

Fig. S1

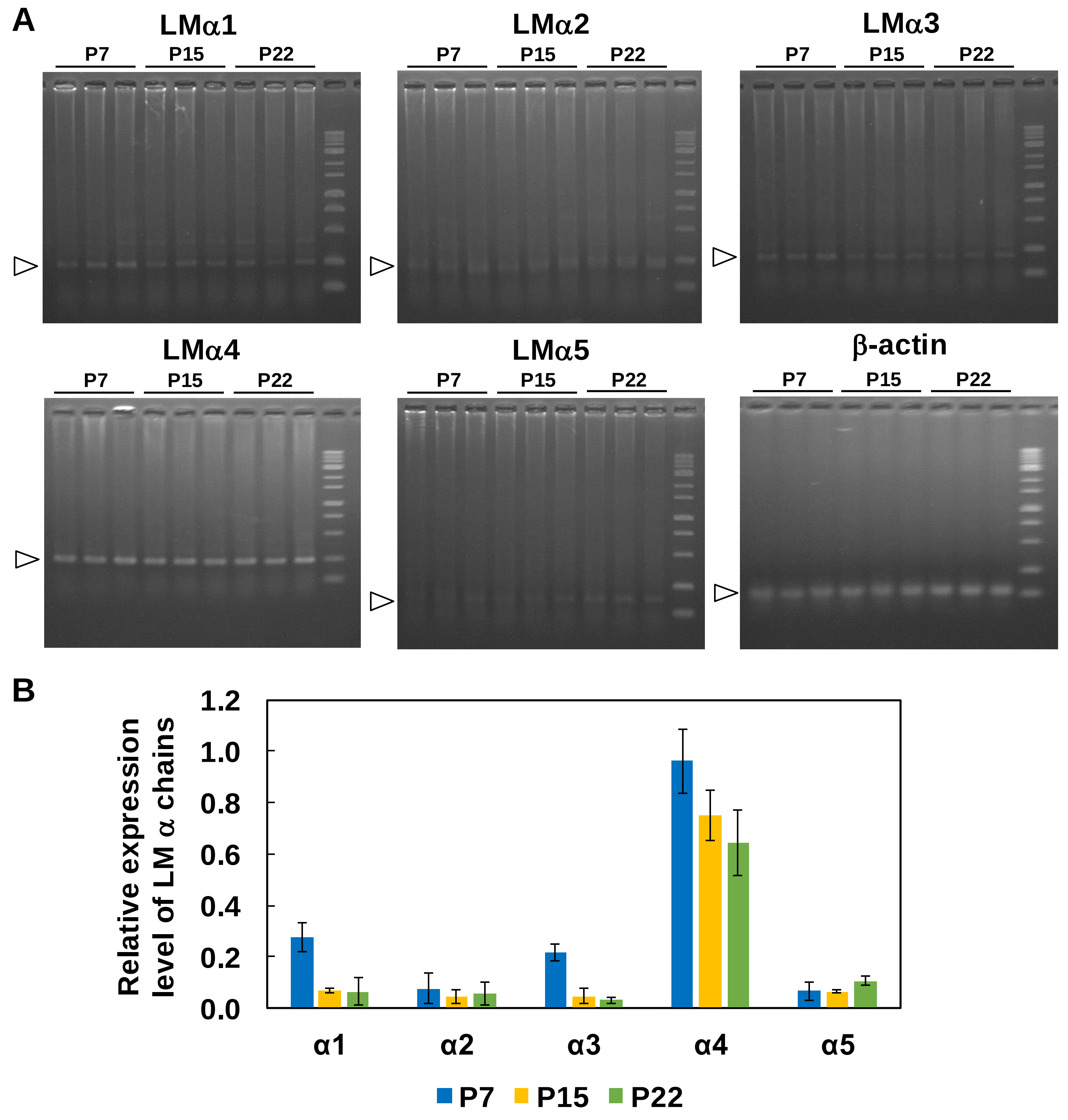

Fig. S1. The mRNA expression levels of LM α chains in wild-type mouse brain. (A) Semi-quantitative reverse transcription-PCR of LM α chains from 3 individual mouse brains at the age of P7, P15, and P22. The experimental procedure was performed as previously described (Yamada et al., 2025). The primers for PCR were used as following: LMα1 (Lama1) forward: AACGACCTGGATTCTGTTGG, reverse: TCAGCGTTCACCTTCTGATG; LMα2 (Lama2) forward: GCCTGCCAACTCTGAGAAAC, reverse: TCAATAGACACAGCGGCAAG; LMα3 (Lama3) forward: GGGTGTGACCAAAAAGTGCT, reverse: CATCTTCCAGGGTGACCAGT; LMα4 (Lama4) forward: GACACGTGACCGACATGAAC, reverse: GACAAGATGGGTTGCTTGGT; LMα5 (Lama5) forward: TACAACCTGTGACCCGACAA, reverse: GGAGCCTGTGTCTCAGGAAG. Arrowhead: an expected size of amplified DNA bands. (B) Quantification of the expression level of each LMα chain normalized with the β-actin expression. The intensity of PCR bands was measured using the Image J software. Error bars represent mean ± s.d.

Fig. S2

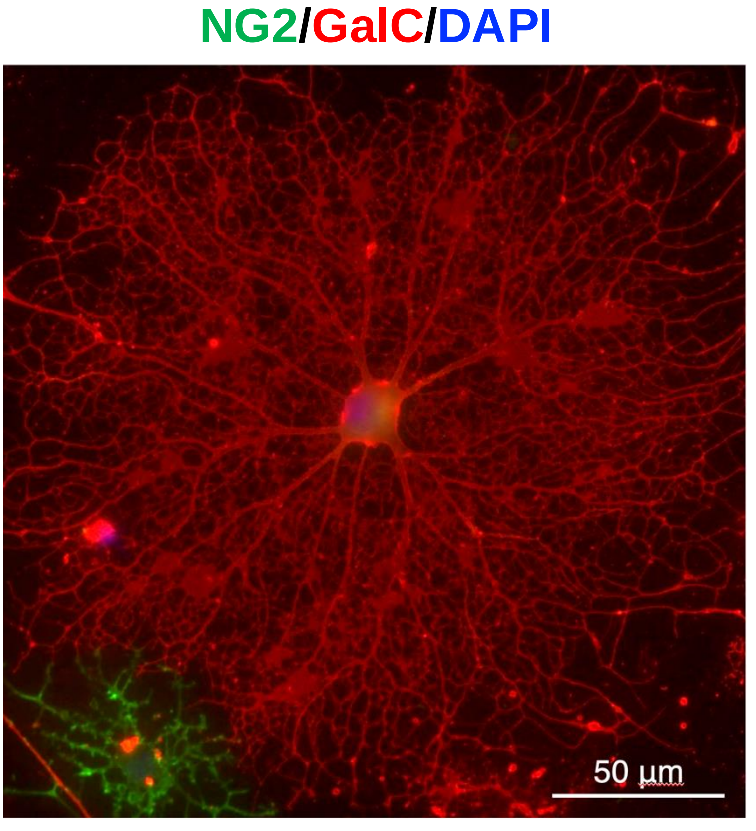

Fig. S2. The effect of LM411 on morphological differentiation of OLs. A representative image of immunocytochemistry of an OL on LM411 is shown (red: GalC; green: NG2). Some of OLs on LM411 exhibited the strikingly morphology with highly branching cell processes, as well as sheet-like myelin membrane formation.

Fig. S3

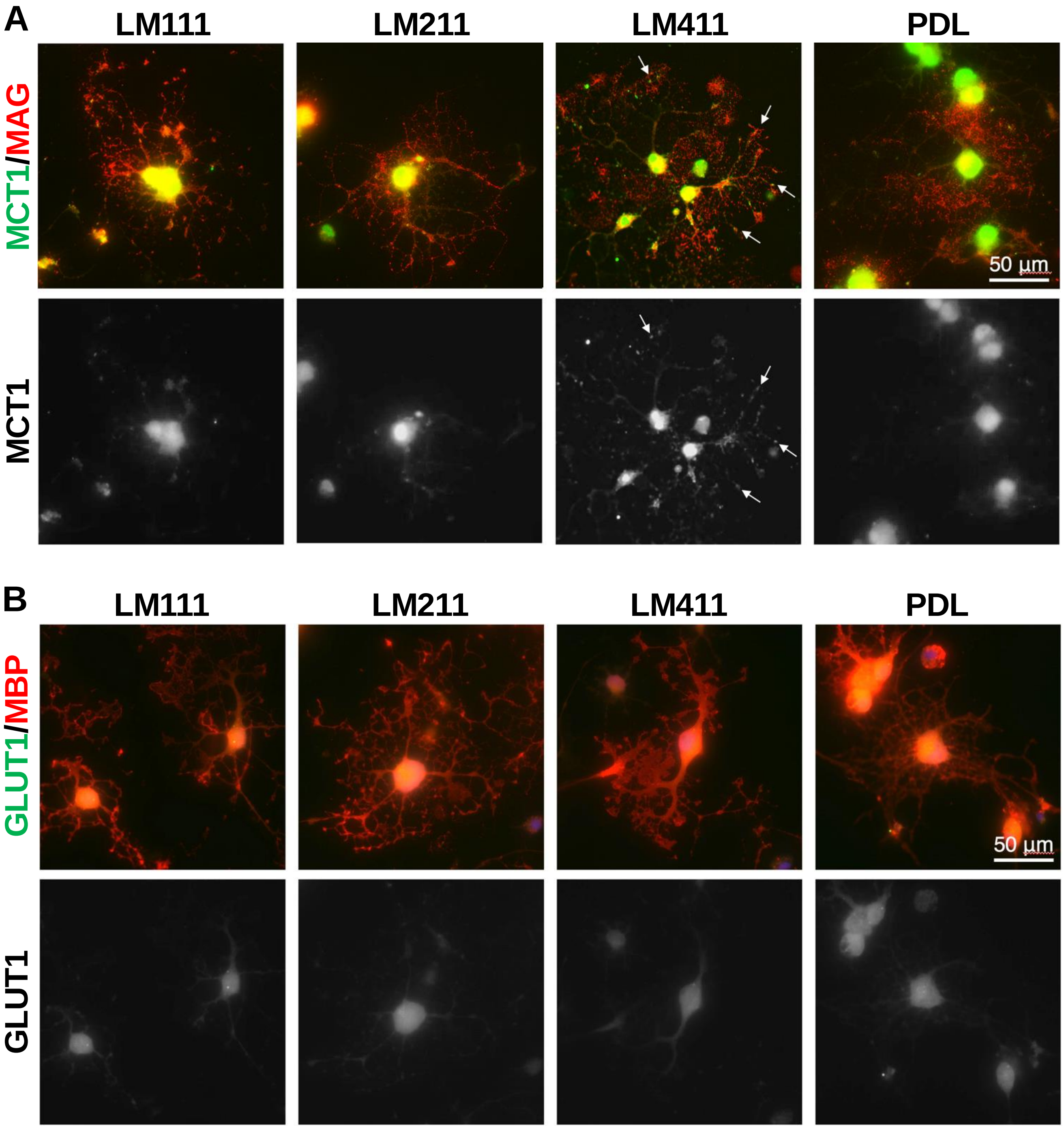

Fig. S3. The effect of LM isoforms on the expression of MCT1 and GLUT1 in OLs. Representative images of immunocytochemistry of (A) MCT1 (green or white) and MAG (red) and (B) GLUT1 (green or white) and MBP (red) are shown. MCT expression was increased in the cell processes of OLs on LM411. Arrows denote the expression of MCT1 at a tip of a process.

Fig. S4

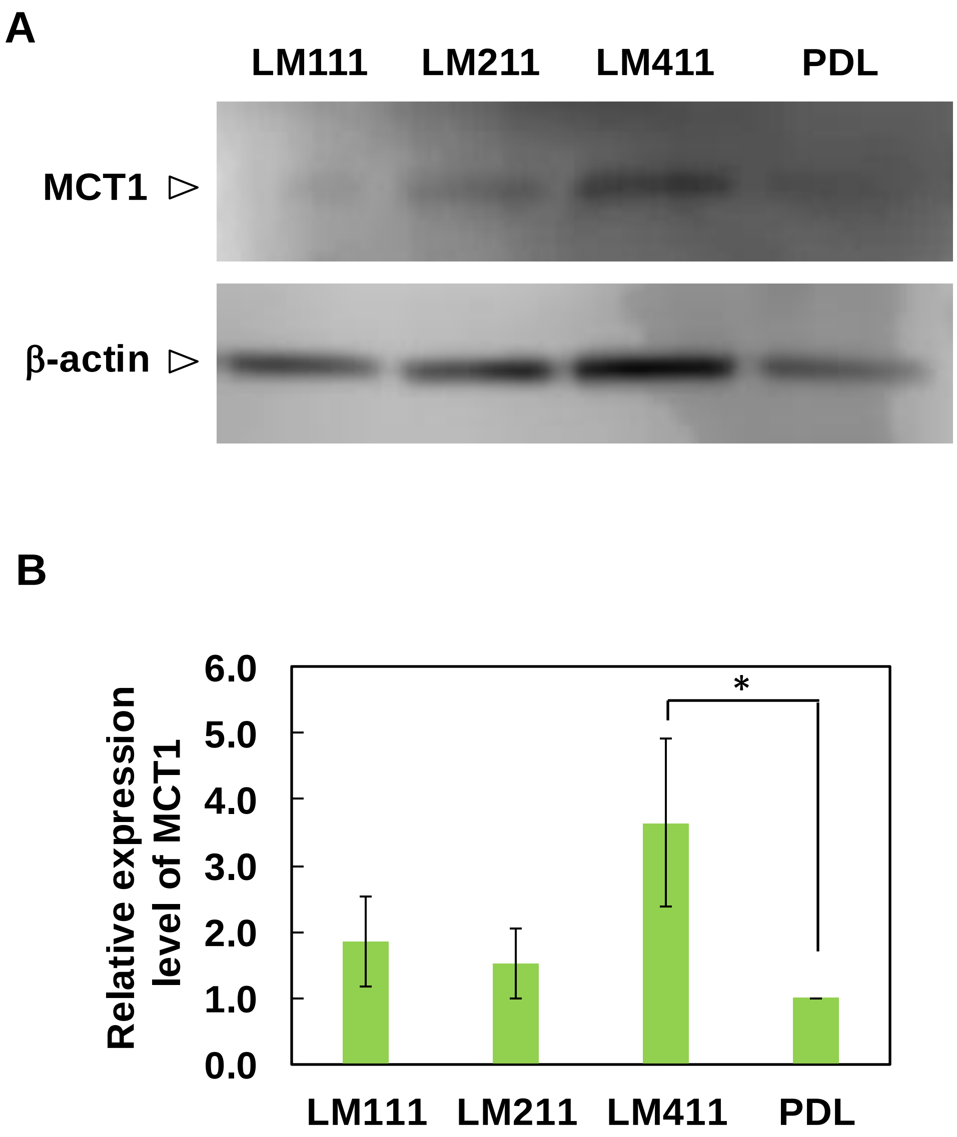

Fig. S4. The increased expression of MCT1 in OLs on LM411. Representative images of (A) Western blotting of MCT1 and β-actin in OLs on LM isoforms and (B) quantification of the Western blotting analysis are shown. The intensity of the protein bands was measured using the Image J software. An increase of the expression of MCT1 in OLs on LM411 was observed. Error bars represent mean ± s.d. (*: $p < 0.05$, n=3; Tukey test).
